# Doppler assessment of aortic stenosis: a 25-operator study demonstrating why reading the peak velocity is superior to velocity time integral

**DOI:** 10.1093/ehjci/jex218

**Published:** 2018-01-15

**Authors:** Stefania Sacchi, Niti M Dhutia, Matthew J Shun-Shin, Massoud Zolgharni, Nilesh Sutaria, Darrel P Francis, Graham D Cole

**Affiliations:** Imperial College London, NHLI—Cardiovascular Science, Du Cane Road, London, UK

**Keywords:** echocardiography, valvular heart disease

## Abstract

**Aims:**

Measurements with superior reproducibility are useful clinically and research purposes. Previous reproducibility studies of Doppler assessment of aortic stenosis (AS) have compared only a pair of observers and have not explored the mechanism by which disagreement between operators occurs. Using custom-designed software which stored operators’ traces, we investigated the reproducibility of peak and velocity time integral (VTI) measurements across a much larger group of operators and explored the mechanisms by which disagreement arose.

**Methods and results:**

Twenty-five observers reviewed continuous wave (CW) aortic valve (AV) and pulsed wave (PW) left ventricular outflow tract (LVOT) Doppler traces from 20 sequential cases of AS in random order. Each operator unknowingly measured each peak velocity and VTI twice. VTI tracings were stored for comparison. Measuring the peak is much more reproducible than VTI for both PW (coefficient of variation 10.1 vs. 18.0%; *P* < 0.001) and CW traces (coefficient of variation 4.0 vs. 10.2%; *P* < 0.001). VTI is inferior because the steep early and late parts of the envelope are difficult to trace reproducibly. Dimensionless index improves reproducibility because operators tended to consistently over-read or under-read on LVOT and AV traces from the same patient (coefficient of variation 9.3 vs. 17.1%; *P* < 0.001).

**Conclusion:**

It is far more reproducible to measure the peak of a Doppler trace than the VTI, a strategy that reduces measurement variance by approximately six-fold. Peak measurements are superior to VTI because tracing the steep slopes in the early and late part of the VTI envelope is difficult to achieve reproducibly.

## Introduction

Aortic stenosis (AS) is a common valvular heart disease^1^ in which echocardiographic assessment is a key part of judgment of timing procedural intervention.^2^^,^^3^ Although other imaging modalities can provide useful complementary information,^4^ echocardiography is the standard technique for serial monitoring.^2^ Nevertheless, it can be challenging to avoid random variation between assessments on separate visits.

Echocardiography provides a range of parameters^5–8^ including peak aortic velocity, aortic velocity time integral (VTI) and, by using measurements of the left ventricular outflow tract (LVOT) and the continuity equation, aortic valve (AV) area. Dimensionless index (DI) is calculated as the Doppler measurement made in the LVOT divided by the Doppler measurement made at the AV. It avoids measurements of the size of the LVOT.^5–8^

One of the many sources of variation between visits is differences that arise between different observers reading the same trace. Previous studies have evaluated the reproducibility of peak and VTI measurements in patients with AS.^9–12^ However, only two operators were studied. Whether the results from a single pair of operators can be generalized to a larger group of operators is unknown.

Moreover, computer technology now allows research to probe deeper into the causation of variability in human measurement processes, in order to provide mechanistic information to those developing clinical protocols to perform efficiently and consistently.

In this study, we asked 25 operators to make measurements from 40 cases, which, unbeknown to operators, were 20 cases shown twice, enabling assessment of intra-operator reproducibility. The aims of our study were to quantify, across a broader range of operators and cases of AS, the intra-operator and inter-operator reproducibility of measurements made from Doppler traces.

## Methods

We reviewed our clinical imaging database to identify 20 consecutive patients undergoing transthoracic echocardiography in the Echocardiography Department at St. Mary’s Hospital in which AS of any severity [defined as peak continuous wave (CW) Doppler velocity of greater than 2 m/s across AV] had been identified.

All patients had undergone standard Doppler examination of flow in the LVOT and AV as recommended by guidelines.^7^^,^^8^ Images were acquired by accredited echocardiographers who were free to optimize sweep speed, scale, gain, and filters as they wished.

The LVOT and AV Doppler trace images for each patient were exported and anonymized. We used custom-designed software (Matlab and Statistics Toolbox Release Matlab R2015b, The MathWorks, Inc., Natick, MA, USA) to mask all but one beat. Whilst in normal clinical practice operators would be free to make measurements from any beat, we wanted to specifically study the process of measuring from the trace to specifically compare peak and VTI. To enable assessment of intra-operator as well as inter-operator variability, the 40 images (20 CW and 20 PW images) were then duplicated (creating 80 images in total) and presented in random order.

Twenty-five operators from three different hospitals, unaware of the duplication and blinded to the results of their or others’ measurements and study hypothesis, were asked to view the images. For each image, they were asked to measure both the peak velocity and the VTI using custom-designed software. The VTI traces were stored for comparison.

### Reversal of traces for VTI

To further investigate variability in tracing the VTI, we attempted to isolate the variability arising from (i) the challenge facing an operator when deciding where to begin a trace and (ii) the challenge of tracing the steep gradients that arise at both the beginning and end of the trace. To do this, images were reversed along the horizontal axis, so that the beginning of the trace was now at the end and vice versa. Ten operators unware of the image reversal, or the reasons for it, were asked to review the 80 reversed images and again make measurements of VTI.

### Statistical analysis

Statistical analysis was performed using ‘The R project for statistical computing’ with the package lme4.^13^ Figures were prepared using the package ggplot2.^14^ Continuous data are expressed as mean ± standard deviation. Categorical variables are summarized as percentages. A *P*-value of <0.05 was considered significant. To quantify intra- and inter-observer variability, we use a mixed-model analysis.^13^ To calculate the variability as a percentage of the measurement, we calculated the variability of the log transformed measurement and then back-transformed the variance.

For each measurement, the percentage difference, scaled to the mean of all operators’ measurements, was calculated to quantify the tendency for individual operators to over-read or under-read.

To quantify the sources of variability arising from tracing a VTI, mean consensus curves were identified and divided into five equal vertical strips. For each strip, the mean standard deviation from the consensus curve was calculated.

Analysis of variance (ANOVA) and the post-hoc Tukey Honest Significant Difference test were used to perform a comparison between the mean variability at different segments of the tracings.

## Results

### Cases

The average age of patients was 79 ±10 years. Eight (40%) were male and 12 (60%) were female. The severity (as described by the reporting physician) was mild in 1 (5%), moderate in 12 (60%), and severe in 7 (35%) patients. The indications for echocardiography were: AS follow-up in 12 (60%) patients, to investigate the cause of shortness of breath in 5 (25%) patients, to investigate the presence of a systolic murmur in 2 (10%) patients, and preoperative evaluation in 1 (5%) patient.

### Operator characteristics

Twenty-five operators from our institution reviewed 80 Doppler images in random order. These consisted of 40 pairs of images: 20 continuous-wave traces acquired through the AV and 20 paired pulsed-wave traces acquired from the LVOT. In fact, the 40 pairs were 20 pairs shown twice, but operators viewing the sequence of 80 randomly ordered traces were unaware of this duplication. Mean experience of echocardiography was 5.8 ± 6.4 years. Twelve (48%) held formal accreditation.

### Variability in VTI and peak measurements

**Figure 1:**
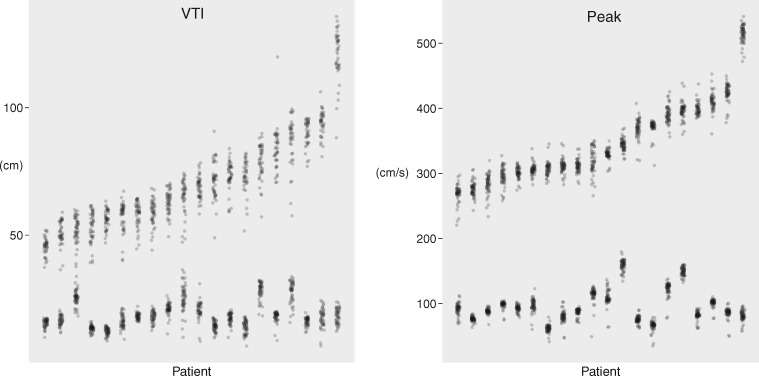
Variation in VTI (left panel) and peak (right panel) measurements. Each column represents a different case, ordered from the smallest average measurement on the left to largest on the right. Each point represents an operator’s measurement for that case. The upper group are measurements from a CW acquisition through the AV. The lower group are measurements from a pulsed-wave acquisition in the LVOT.

The distribution of VTI measurements is shown in the left panel of *Figure **1*. Across all measurements made by all operators in all cases, the overall mean VTI was 70.1 ±18.6 cm for CW through the AV and 18.7 ± 4.7 cm for PW in the left ventricular tract. Across all cases, the coefficient of variation was 18.0% for pulsed-wave Doppler traces in the LVOT, made up of an intra-operator coefficient of variation of 11.9% and an inter-operator coefficient of variation of 12.9%. Across all cases, the coefficient of variation was 10.2% for CW Doppler traces through the AV, made up of an intra-operator coefficient of variation of 7.3% and an inter-operator coefficient of variation of 6.9%.

The distribution of peak measurements is shown in the right panel of *Figure **1*. Across all measurements made by all operators in all cases, the overall mean peak velociy was 346.5 ± 62.0 cm/s for CW through the AV and 95.8 ± 25.0 cm/s for PW in the left ventricular tract. Across all cases, the coefficient of variation was 10.1% for pulsed-wave Doppler traces in the LVOT, made up of an intra-operator coefficient of variation of 5.6% and an inter-operator coefficient of variation of 8.2%. Across all cases, the coefficient of variation was 4.0% for CW Doppler traces through the AV, made up of an intra-operator coefficient of variation of 2.5% and an inter-operator coefficient of variation of 3.1%.

As can be seen from *Figure **1*, peak values were more tightly clustered than VTI values. The coefficient of variation was significantly smaller (*P* < 0.001 by ANOVA, and the post-hoc Tukey Honest Significant Difference test).

### Tendency for an operator to over-read or under-read on repeated viewing of identical images

**Figure 2:**
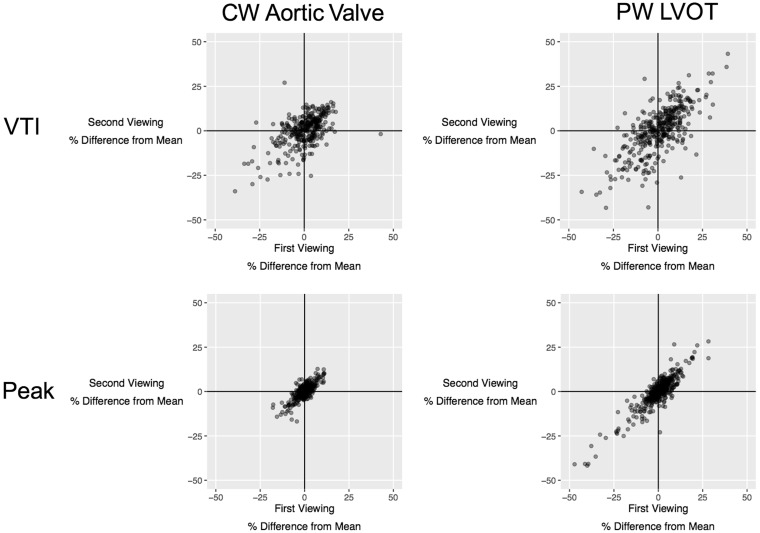
Consistency in operators’ behaviour when reassessing the same images. Each point represents a measurement made by one operator viewing one case. The position on the horizontal axis represents whether the operator over-read or under-read on the first viewing and scaled to the average of all operators for that case. The position on the vertical axis represents whether the operator over-read or under-read on the second viewing and again scaled to the average of all operators for that case. Operators consistently over-reading on both viewings lie in the top-right quadrant, whereas operators consistently under-reading on both viewings lie in the bottom-left quadrant. The upper panel shows measurements of VTI. The lower panel shows measurements of peak velocity. The left panel shows pulsed-wave LVOT measurements. The right panel showed CW AV measurements.

Across all measurements, operators showed a tendency to consistently make measurements which over-read or under-read the average of all operators when they were unknowingly represented with the same image again, as shown in *Figure **2*. Proportional over-measurement or under-measurement was strongly correlated for first and second VTI measurements of AV CW traces (Pearson’s *r *= 0.48; *P* < 0.001). It was also strongly correlated for first and second VTI measurements of LVOT PW traces (*r* = 0.78; *P* < 0.001). Even stronger correlations were observed for first and second peak measurements of AV CW traces (Pearson’s *r* = 0.78: *P* < 0.001) and first and second peak measurements of LVOT PW traces (Pearson’s *r* = 0.89: *P* < 0.001).

### Tendency for an operator to over-read or under-read in general for any case

**Figure 3:**
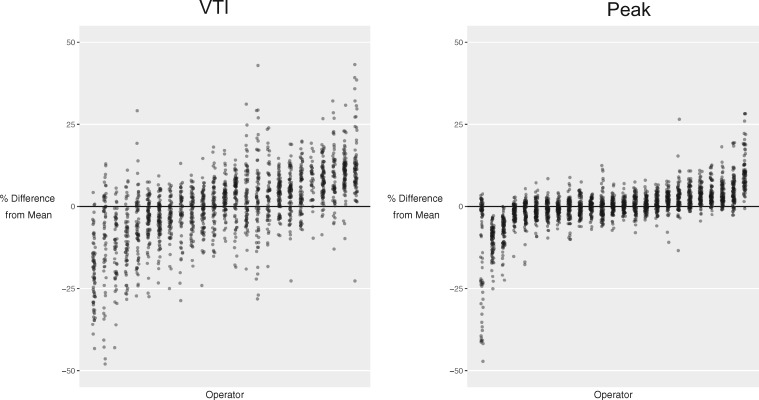
Tendency of operators to under-read or over-read relative to the average for that case. Each column represents a different operator, ordered from the operator under-reading by the largest proportion on the left to the operator over-reading by the largest proportion on the right. The values have been scaled to the average for that case. The left panel shows VTI measurement. The right panel shows peak measurement.

When an operator’s measurements of all the cases were considered, some operators had a tendency to under-read and over-read across cases in general. For VTI, the operator with the largest tendency to under-read did so by −19.6 ± 10.2%, whilst the operator with the largest tendency to over-read did so by +12.8 ± 10.4%. For peak, the operator with the largest tendency to under-read did so by −12.6 ± 15.9%, whilst the operator with the largest tendency to over-read did so by +10.8 ± 6.3%. The distribution of the tendency to under-read or over-read by individual operators is shown in *Figure **3*.

### Tendency to over-read and under-read CW and PW images from the same case

**Figure 4:**
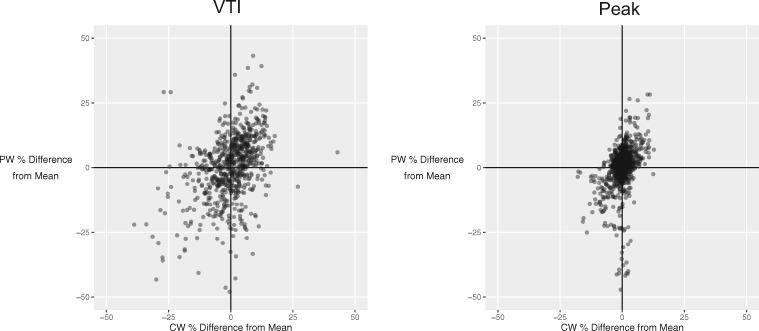
Relationship between under-reading and over-reading for pulsed-wave and continuous wave traces from the same patient for VTI (left panel) and peak measurements (right panel). Each point represents a case reviewed by a single operator. The tendency to over-read or under-read the pulsed wave LVOT measurement is represented on the horizontal axis. The tendency to over-read or under-read the continuous wave AV measurement is represented on the vertical axis.

When considering an individual patient, an operator making an AV CW VTI measurement higher than the average from all operators was also likely to make an LVOT VTI measurement higher than the average from all operators (Pearson’s *r *= 0.39; *P* < 0.001) (*Figure **4*, left panel). Similarly, operators making an AV CW peak velocity measurement higher than the average from all operators were also likely to make a LVOT PW peak velocity measurement higher than the average from all operators (Pearson’s *r *= 0.41; *P* < 0.001) (*Figure **4*, right panel).

### Variability in dimensionless index measurements

Across all measurements made by all operators in all cases, the overall mean DI was 0.280 ± 0.077 and 0.282 ± 0.080 using VTIs and peak velocities, respectively. Across all cases, the coefficient of variation was 9.3% for DI using peak, made up of an intra-operator coefficient of variation of 6.2% and an inter-operator coefficient of variation of 6.7%. Across all cases, the coefficient of variation was 17.1% for DI using VTI, made up of an intra-operator coefficient of variation of 13.9% and an inter-operator coefficient of variation of 9.3%.

### Steep slopes are the main source of VTI tracing variability

**Figure 5:**
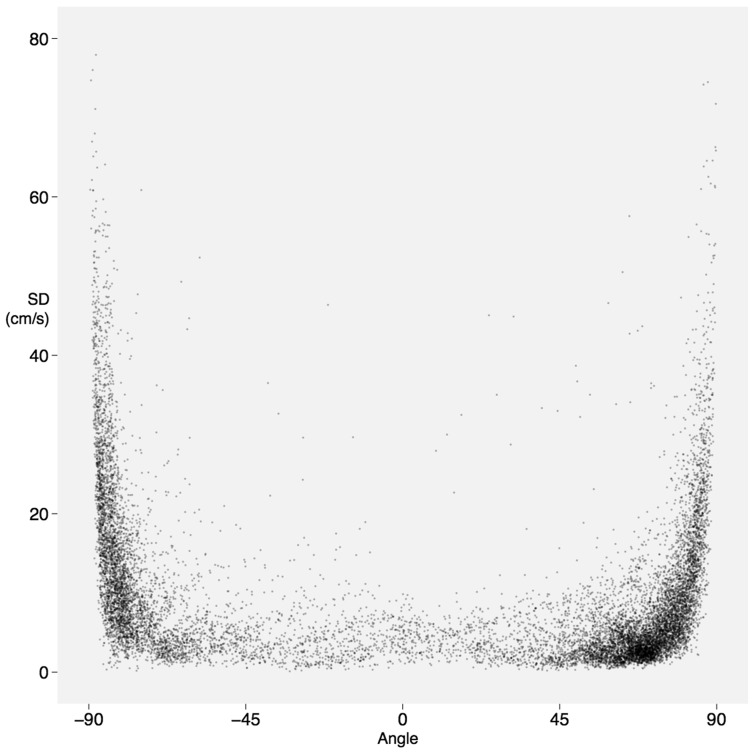
Disagreement with the consensus of all operators at steep angles. Each point represents a small portion of an individual operator's trace for an individual case. Deviation from the consensus is low at shallow angles but far greater when the slope is steep, whether it be downwards from the baseline (negative angle, left of the diagram) or back towards the baseline (positive angle, right of the diagram).

The variability in tracing followed the same pattern for both CW and pulsed wave (PW) Doppler traces, as shown in *Figure **5*. The standard deviation of the trace from the consensus is low for segments of the trace which have either no or shallow slopes. The standard deviation becomes much higher when the slopes are steeper.

**Figure 6:**
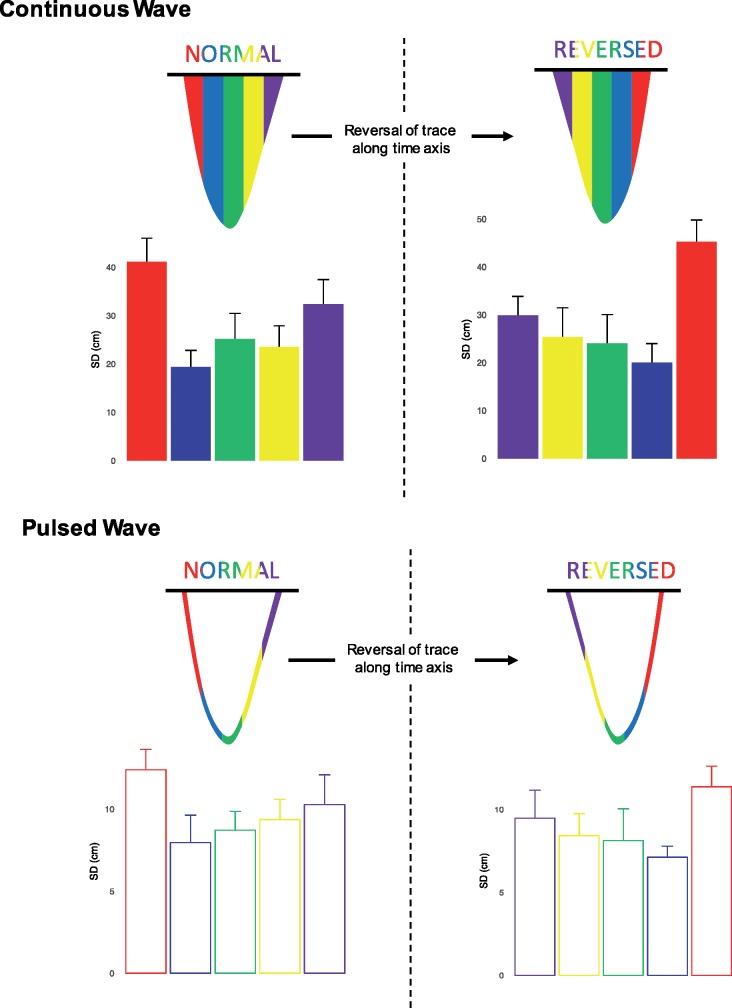
Variation arising from tracing of continuous wave (upper panels) and pulsed-wave (lower panels) velocity time integral. Each beat is divided into five columns of equal width. The variability is highest in the columns at the beginning and end of the traces. The left panels show the standard deviation for traces presented normally. The right panels show the standard deviation for traces when the horizontal (time) axis is reversed.

When variability is considered across five equal vertical sections of the trace (as shown in *Figure **6*), the highest variability was seen in the first part of the trace [standard deviation (SD) 41.1 ± 12.6 cm for CW, SD 12.4 ± 2.3 cm for PW]. The lowest variability was seen in the middle parts of the trace. The last part of the trace showed intermediate variability (SD 32.4 ± 9.5 cm for CW, SD 10.3 ± 4.7 cm for PW).

### Steep slopes rather than initiating the trace is the source of variability

To test the hypothesis that variability arises from the difficulty in reliably tracing the steep part of the curve rather than the act of deciding where to begin the trace, we represented the images to 10 observers a third and a fourth time, but with the images flipped horizontally (i.e. the time-axis reversed).

When tracings were reversed (right panels on *Figure **6*), the trend in variability was also reversed. The highest variability was seen in the last part of the trace (SD 45.3 ± 12.2 cm for CW, SD 11.4 ± 2.3 cm for PW). The lowest variability was again seen in the middle part of the trace. The first part of the trace showed intermediate variability (SD 29.9 ± 7.4 cm for CW, SD 9.5 ± 4.1 cm for PW).

## Discussion

This study shows that measuring the peak of a Doppler trace is a far more reproducible strategy than measuring the VTI, with, on average, a 2.5-fold reduction in coefficient of variation. In research, the resulting six-fold reduction in the number of patients required to power a study using peak velocity rather than VTI has huge financial and logistical implications. In clinical practice, a patient with the average peak velocity from our study of 346.5 cm/s, a change of 38.5 cm/s (11.1%) could be detected with 95% confidence. In clinical practice, a patient with the average VTI from our study of 70.1 cm, a change of 19.8 cm (28.3%) could be detected with 95% confidence.

The management of AS depends on accurate quantification of severity.^2^^,^^3^ The AV and LVOT VTIs are routinely used to calculate aortic valve area (AVA) by the continuity equation, but peak velocities are often substituted^2^ based on the evidence that both AVA and DI, derived interchangeably from either VTIs or peak velocities, correlated well with the gold standard catheterization-derived AV area.^15–22^ However, in order to be clinically useful, a parameter must be accurate and reproducible.^5^^,^^6^ In this study, we show that peak velocity is considerably more reproducible than VTI.

### Comparison with previous studies

The reproducibility of peak velocity and VTI has been previously studied with two operators.^9–12^ Just as a study that attempted to assess the average height of a population would measure more than two people, a study measuring the average performance of operators should ideally measure more than two operators. Our study is unique in testing reproducibility across a much larger group. The other benefit of measuring more than two operators is that it is worthwhile setting up a data collection system that allowed operators to make blinded reassessments, allowing us to study both intra-operator and inter-operator reproducibility and the mechanism of disagreement when tracing a VTI.

We found that the CW aortic peak velocity was more reproducible, with an intra-operator and inter-operator variability of 2.5% and 3.1% leading to an overall coefficient of variation of 4.0%. This is consistent with the values previously reported.^9–12^ Our results for PW LVOT peak velocity were less reproducible than the previous literature^10^ with an intra-operator and inter-operator variability of 5.6% and 8.2%, respectively leading to an overall coefficient of variation of 10.1%. CW VTI had intra-operator and inter-operator variability of 7.3% and 6.9% leading to an overall coefficient of variation of 10.2%. For PW VTI, this study shows worse reproducibility than previous studies; PW VTI had intra-operator and inter-operator variability of 11.9% and 12.9% leading to an overall coefficient of variation of 18.0% which is higher than the intra-operator and inter-operator variability previously demonstrated.^10^

### A reason why dimensionless index works: systematic under-reading or over-reading of both AV and LVOT traces by individual operators

This study confirms that DI shows better reproducibility across operators than would be expected from two peak or VTI measurements made in isolation. Some operators demonstrated a tendency to consistently make measurements which were smaller than or larger than the average for that case, as shown in *Figure **3*. When they interpret PW and CW images from the same patient, they show a consistent tendency to make measurements smaller than or larger than the average for that image, as shown in *Figure **4*. This is important, because it underlies some of the benefit of DI, which arises because an under-read in one image matched by an under-read in the other image will tend to cancel out and lead to an comparatively consistent DI.

**Figure 7:**
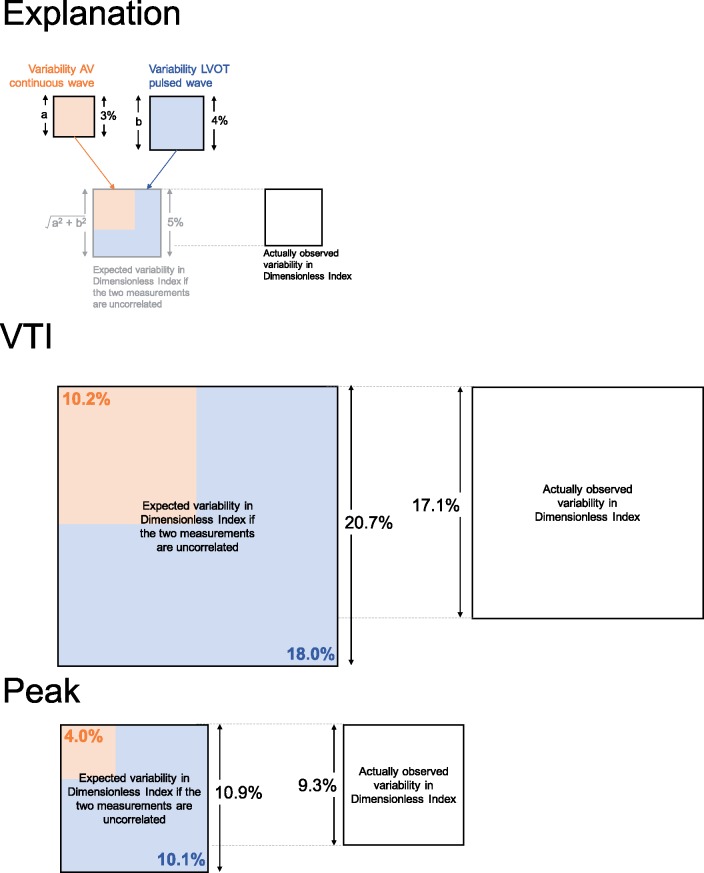
Expected and observed proportional variance in dimensionless index. The area of the square represents the proportional variance. The orange area is the CW AV variance and the blue area the pulsed-wave LVOT variance. The top panel shows that the expected variance can be calculated from CW and PW variances (combined orange and blue). The actually observed variance (white) indicates the benefit that arises from dimensionless index. The middle panel shows the benefit of dimensionless index in VTI (coefficient of variation 20.7–17.1%). The bottom panel shows the benefit of dimensionless index in peak measurements (coefficient of variation 10.9–9.3%).

Based on the mathematical principle of propagation of errors, the coefficient variation for DI can be estimated as the square root of the summed squares of the coefficients of variation of the two measurements forming the ratio.^23^ For peak measurements, the coefficient of variation of individual AV and LVOT measurements was 4.0% and 10.1%, respectively. The coefficient of variation of the resulting DI would therefore be expected to be ∼√(4.0^2^ + 10.1^2^), which is 10.9%, but we discovered it to be only 9.3%. Similarly, one might expect the VTI-derived DI based to have a coefficient of variation of 20.7%, but our data showed it to be only 17.1%. As shown in *Figure **7*, DI produces smaller coefficients of variation than would be expected from combining the two measurements.

### Source of variability in tracing VTI

Our analysis shows that most of the noise arising when measuring the VTI occurs at the beginning and the end of the Doppler trace. When compared to the middle of the wave, the variation from the consensus curve is larger at the beginning and end of the wave. Our experiment of reversing the images showed a corresponding reversal in the pattern of variability: it is not the act of deciding where to start tracing, but the steep slope of the Doppler trace which hinders reproducibility.

### Limitations

In this analysis, 25 operators viewed the same images. There is not the same as 25 operators acquiring their own images and then making measurements from them. The variability we demonstrate in this study is a lower limit estimate, since the acquisition of different images would add further variability but could not reduce it. We also selected only one beat for each patient, excluding beat-to-beat variability, which is another reason our result is a lower limit estimate. However, this study indicates that further work to characterize the variability arising from different operators making measurements or different operators choosing different beats should take place using peak rather than VTI measurements.

Assessment of AS severity includes more than Doppler measurements. In the real world, clinicians integrate other imaging findings (such as the morphological appearances of the AV) and clinical information in their assessment. The relationship between the number of different pieces of information provided to operators and variability in their overall assessment of severity remains unknown.

## Conclusions

Measuring the peak of a Doppler trace is a more reproducible strategy than measuring the VTI. The inferiority of VTI reproducibility arises mainly because of disagreement at the beginning and end of the tracing where the slope of the Doppler trace is steep. Individual operators show a tendency to over-read or under-read, which is responsible for some of the benefit of dimensionless index.

The extent of superiority of peak over VTI for an individual patient is non-trivial: an average operator would be 95% sure of detecting a difference of 11.1% difference in peak velocity between two different images. For VTI, the same confidence would only arise with a much larger 28.3% change. Similarly, a clinical trial using a VTI as the endpoint would have to be more than six times larger than one using peak velocity.
